# Siglec-5 suppresses LPS-induced acute lung injury via negative regulation of HSF1/SYK-mediated ROS production and pyroptosis

**DOI:** 10.3389/fmed.2026.1799874

**Published:** 2026-06-30

**Authors:** Jiwei Zhang, Weijiang Bai

**Affiliations:** Department of Trauma Center, The First Hospital of Hebei Medical University, Shijiazhuang, China

**Keywords:** HSF1/SYK/ERK1/2/SIRPα, lung injury, pyroptosis, sepsis, Siglec-5

## Abstract

**Objective:**

To investigate the protective effect of Siglec-5 on lung injury in septic mice and its molecular mechanism.

**Methods:**

Forty-eight male SPF-grade ICR mice were randomly divided into the sham group, Model group (induced by LPS), Siglec5-OE group, and Siglec5-OE + SIRPα-OE group. After modeling, HE staining was used to evaluate pathological changes in lung tissue. ELISA was employed to measure the levels of MDA, SOD, GPX, IL-1β, IL-18, and GSDMD. RAW264.7 cells were treated with drugs, and Western blot was used to detect protein expression. Flow cytometry was performed to assess apoptosis, fluorescent probes were used to measure ROS fluorescence intensity, and immunofluorescence was used to detect NLRP3 fluorescence intensity.

**Results:**

Compared with the sham group, the Model group showed severe lung tissue damage, increased levels of MDA, IL-1β, IL-18, and GSDMD, and decreased levels of SOD and GPX. Compared with the Model group, the Siglec5-OE group exhibited improved lung injury, reduced levels of MDA, IL-1β, IL-18, and GSDMD, and increased levels of SOD and GPX. Compared with the Siglec5-OE group, the Siglec5-OE + SIRPα-OE group showed significantly aggravated lung tissue damage, increased levels of MDA, IL-1β, IL-18, and GSDMD, and decreased levels of SOD and GPX. *In vitro* experiments demonstrated that Siglec-5 promoted HSF1 protein expression, inhibited p-SYK, p-ERK, and SIRPα protein expression, thereby suppressing apoptosis, reducing ROS activity, and inhibiting NLRP3-mediated pyroptosis.

**Conclusion:**

Siglec-5 inhibits LPS-induced lung injury by regulating the HSF1/SYK/ERK1/2/SIRPα signaling pathway, thereby modulating ROS and pyroptosis responses.

## 1 Introduction

Sepsis is a systemic inflammatory response syndrome caused by infection, leading to dysregulation of homeostasis and multiple organ dysfunction. The lungs are among the earliest organs to suffer acute injury, often resulting in mortality (1). Although clinical individualized treatment strategies for sepsis have been gradually optimized, effective specific therapies remain lacking. Multiple clinical studies indicate that the overall survival rate of sepsis has not significantly improved, with mortality rates still as high as 40%, making sepsis a significant challenge in ICU care (2). Therefore, medical research has long focused on investigating the pathophysiology of sepsis and looking for efficient preventive and therapy methods. Acute respiratory distress syndrome (ARDS), which is characterized by alveolar epithelial cell destruction, inflammatory cell infiltration, and pulmonary interstitial edema, is a typical consequence of lung injury in sepsis patients (3). In addition to making sepsis patients’ condition worse, lung damage dramatically raises treatment challenges and mortality. Thus, it is crucial from a clinical standpoint to look into the pathophysiology of sepsis-related lung damage and find possible treatment targets.

According to recent research, immune regulatory molecules are essential in sepsis and the organ damage it causes. Among them, the sialic acid-binding immunoglobulin-like lectin (Siglec) family has attracted interest because of its extensive expression on immune cells and function in controlling inflammatory reactions (4). Siglecs are a type of immunological receptors that bind to glycans containing sialic acid to control the activation and activity of immune cells (5). It has progressively come to light that Siglec-5, a significant member of the Siglec family, is involved in immune modulation and inflammation. Oxidative stress and pyroptosis are thought to be important factors in the pathological process of sepsis-related lung damage, which includes several molecules and signaling pathways (6). The imbalance between the generation and removal of reactive oxygen species (ROS), which causes structural and functional damage to cells, is known as oxidative stress (7). In addition to directly harming DNA and cell membranes, ROS may trigger a number of inflammatory signaling pathways that intensify inflammatory reactions. The NLRP3 inflammasome is the primary mediator of pyroptosis, a planned cell death process marked by cell membrane rupture and the release of inflammatory chemicals (8). Pro-inflammatory cytokines including IL-1β and IL-18 are secreted when the NLRP3 inflammasome is activated, which exacerbates tissue damage (9). Thus, preventing oxidative stress and pyroptosis might be a key tactic for reducing lung damage brought on by sepsis. Heat shock factor 1 (HSF1) is a crucial transcription factor that is involved in cellular stress responses at the molecular level (10). By controlling the production of heat shock proteins (HSPs), HSF1 may shield cells from oxidative stress and inflammatory damage. Furthermore, important signaling molecules implicated in inflammation and immunological responses include extracellular signal-regulated kinase (ERK1/2) and spleen tyrosine kinase (SYK) (11, 12). Pyroptosis and the generation of inflammatory substances are encouraged when the SYK/ERK1/2 signaling pathway is activated. However, by ligand binding, signal regulatory protein alpha (SIRPα), an immunological inhibitory receptor, may prevent immune cell activation.

Based on the above background, this study aimed to explore the protective effect of Siglec-5 on LPS-induced acute lung injury in mice and its molecular mechanisms. Given the high sequence homology and conserved expression of Siglec-5 on myeloid cells between mice and humans, the murine system serves as a relevant model for investigating its role in sepsis. By constructing a sepsis mouse model, we observed the effects of Siglec-5 overexpression on lung tissue pathological changes, oxidative stress levels, and inflammatory factor expression, and further investigated its relationship with the HSF1, SYK, ERK1/2, and SIRPα signaling pathways. Additionally, *in vitro* experiments using RAW264.7 cells examined the regulatory effects of Siglec-5 on apoptosis, ROS production, and NLRP3 inflammasome activation, providing new theoretical insights and potential therapeutic targets for sepsis-related lung injury. This study is the first to explore the interaction between Siglec-5 and SIRPα, offering a new direction for in-depth research on immune regulatory molecules in sepsis.

## 2 Materials and methods

### 2.1 Experimental animals

Overexpression study: This study used 48 male SPF-grade ICR mice, aged 6–8 weeks, weighing 30 ± 5 g. The mice were provided by Henan Sikebeisi Experimental Animal Company (License No.: SCXK (Yu) 2021–0009). The mice were housed at 22 ± 2 °C with a 12-h light–dark cycle, with free access to food and water. After a 7-day acclimatization period, the experiment was conducted. The mice were randomly divided into four groups: sham group, Model group, Siglec5-OE group, and Siglec5-OE + SIRPα-OE group (*n* = 12/group). Lipopolysaccharide (LPS) powder was dissolved in saline to prepare a 1 mg/mL solution, stored in the dark, and prepared immediately before use to maintain efficacy. The Model group was intraperitoneally injected with LPS at a dose of 0.1 mL/10 g body weight to induce sepsis. The Siglec5-OE and Siglec5-OE + SIRPα-OE groups were injected with the corresponding lentivirus via the tail vein one week before LPS injection. The Siglec5-OE and Siglec5-OE + SIRPα-OE groups received the corresponding overexpression lentivirus via tail vein injection 7 days before LPS challenge. The sham group received equivalent volumes of saline or control lentivirus at the same time points.

Knockdown study: An additional cohort of 24 male SPF-grade ICR mice (same source, age, and housing conditions) was used specifically for Siglec5 loss-of-function experiments. After acclimatization, these mice were randomly divided into three groups: sham group, Model group, and Siglec5-KD group (*n* = 8/group). In the Siglec5-KD group, each mouse received a single tail-vein injection of lentivirus expressing shRNA targeting Siglec5 (1 × 10^8^ TU in 100 μL) 7 days before LPS administration.

### 2.2 Experimental materials

RAW264.7 cells were purchased from Shanghai Bohu Biotechnology Co., Ltd. Isoflurane was purchased from Beijing Keyue Huacheng Technology Co., Ltd., China. LPS was purchased from Sigma, USA. Antibodies against NOX4, Siglec5, SIRPα, HSF1, and NLRP3 were purchased from Cell Signaling Technology, USA. Antibodies against p-SYK, p-ERK, p-ELK1, p-SHP2, IL-1*β*, and GSDMD were purchased from Abcam, UK. β-Actin antibody was purchased from Thermo, USA. HSF1 inhibitor Rocaglamide, SYK inhibitor Piceatannol, ERK1/2 activator mSIRK, and ELK1 inhibitor TAT-DEF-Elk-1 (TDE) were purchased from MCE.

Lentivirus for Siglec5 knockdown: Lentiviral particles encoding shRNA specifically targeting mouse Siglec5 and a non-targeting scrambled control shRNA (shNC) were constructed and packaged by VectorBuilder. The viral titer was 1 × 10^9^TU/mL, and each mouse in the Siglec5-KD group received 100 μL of shSiglec5 lentivirus (1 × 10^8^ TU) via the tail vein. Overexpression lentiviruses for Siglec5 and SIRPα were similarly prepared and used as previously described.

### 2.3 Bioinformatics analysis

To comprehensively investigate the molecular mechanisms of sepsis-induced lung injury, we integrated transcriptomic data from both systemic and local perspectives. The sepsis peripheral blood dataset GSE28750 (10 patients and 20 healthy controls) and the sepsis-related ARDS lung tissue dataset GSE10361 (6 ARDS patients and 3 healthy controls) were retrieved from the GEO database. The goal of this integration is to find “core” regulatory elements that are often dysregulated at both the injury site (which represents local organ pathology) and the systemic circulation (which reflects the systemic immune response), hence revealing possible important disease mediators. However, we recognize that since different tissues have different cellular compositions, direct comparisons between them are limited. The Robust Multi-array Average (RMA) technique was used for probe summarization, quantile normalization, and background correction during data preparation. The ComBat method was used to eliminate batch effects across platforms. Using the limma software, differential expression analysis was carried out. To guarantee multiple hypothesis testing control, screening criteria were established as |log2 Fold Change| > 1 and Benjamini-Hochberg-adjusted *p* < 0.05. Venny 2.1 was used to display the intersection of differentially expressed genes from the two datasets. The ClusterProfiler program was used to do hierarchical functional analysis: KEGG pathway analysis concentrated on important biological signaling networks, while Gene Ontology (GO) enrichment encompassed cellular components (CC), molecular functions (MF), and biological processes (BP). Gene interaction patterns were investigated using Spearman rank correlation coefficient matrices. In order to thoroughly unveil the fundamental molecular underpinnings of sepsis-related lung damage, ggplot2 was used to create multi-level visualizations such as correlation heatmaps, gene set enrichment bubble plots, and differential expression volcano plots.

### 2.4 Quantitative real-time PCR (RT-qPCR)

Total RNA was extracted from the collected cell and tissue samples using TRIzol reagent and subsequently reverse-transcribed into cDNA. Quantitative PCR was then performed using the SYBR Green method. The primer sequences targeting the genes of interest (NLRP3, SYK, GSDMD) and the reference genes (GAPDH) are listed in Supplementary file 1.

Initial denaturation at 95 °C for 30 s, 40 cycles of denaturation at 95 °C for 5 s, and annealing/extension at 60 °C for 30 s comprised the thermal cycling conditions. The amplification specificity was then confirmed via a melting curve study. The 2^−ΔΔCt2^ technique was used to determine the relative mRNA expression levels. Each experiment was carried out three times independently and with three technical replicates per sample. One-way analysis of variance (ANOVA) or Student’s t-test were used to establish statistical significance; a *p*-value < 0.05 was deemed statistically significant.

**Figure 1:**
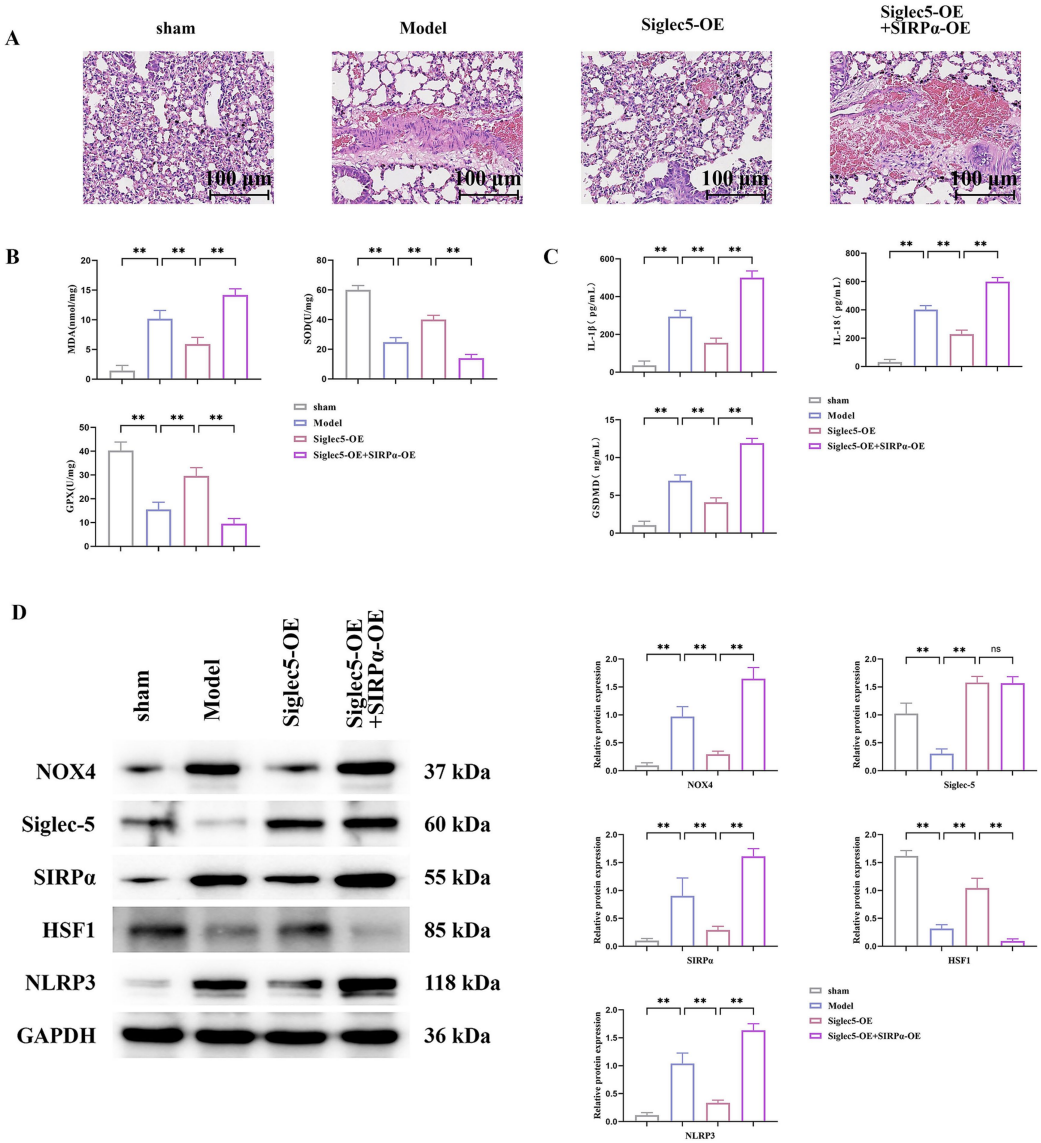
Siglec-5 inhibits LPS-induced lung injury. **(A)** HE staining for assessing lung tissue pathological changes. **(B)** Measurement of MDA, SOD, and GPX levels using assay kits. **(C)** ELISA for measuring IL-1β, IL-18, and GSDMD levels. **(D)** Western blot for detecting NOX4, Siglec5, SIRPα, HSF1, and NLRP3 protein expression. Quantitative analysis was performed using ImageJ software, with values normalized to the GAPDH loading control (*n* = 7).

**Figure 2:**
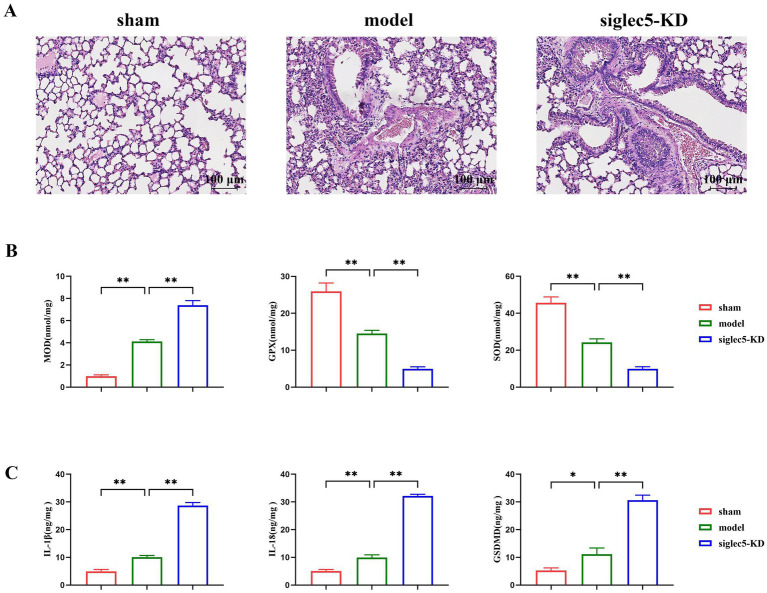
Siglec-5 knockdown exacerbates LPS-induced lung injury and oxidative stress. **(A)** HE staining for assessing lung tissue pathological changes. **(B)** Measurement of MDA, SOD, and GPX levels using assay kits. (**C)** ELISA for measuring IL-1β, IL-18, and GSDMD levels. Data are presented as mean ± SD. *p < 0.05, **p < 0.01 (*n* = 7).

The efficiency of Siglec5 overexpression (Siglec5-OE group, Figure 1) and knockdown (Siglec5-KD group, Figure 2) was verified by RT-qPCR on lung tissues using Siglec5-specific primers (Supplementary file 1). The results are shown in Supplementary Figure S1. Only mice showing ≥70% reduction in Siglec5 mRNA expression were included in the final analysis.

### 2.5 Single-cell transcriptome analysis

#### 2.5.1 Acquisition and preprocessing of single-cell transcriptome data

Two publicly accessible mouse lung single-cell transcriptome datasets (GEO accession: GSM8953943 and GSM8458437) were combined in this investigation. Data quality control was performed using the Seurat v4 R package with the following filtering criteria: mitochondrial gene percentage <20%, ribosomal gene percentage 5–60%, and the number of detected genes per cell (nFeature_RNA) between 200 and 6,000. Batch effects were removed using the FindIntegrationAnchors (default parameters, based on the CCA algorithm) and IntegrateData functions, yielding an integrated expression matrix for downstream analyses.

#### 2.5.2 Cell clustering, annotation, and visualization

**Figure 3:**
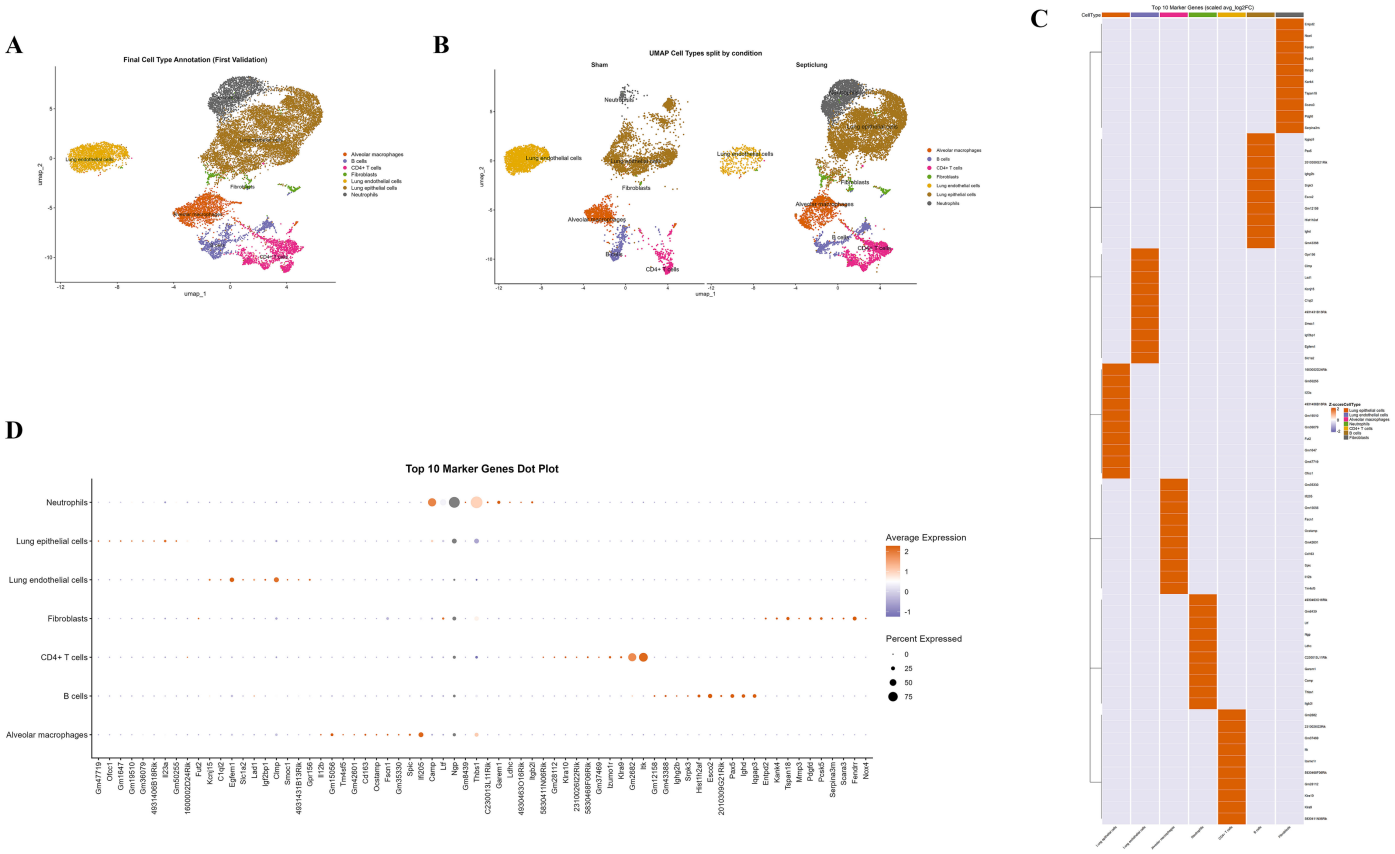
Construction of the lung single-cell atlas and identification of cell subpopulations. **(A)** UMAP plot of all cells, displaying seven major cell types. **(B)** UMAP plot colored by experimental group (Sham vs. Sepsis). **(C)** Heatmap of the top 10 marker genes for each cell subpopulation. **(D)** Dot plot of the top 10 marker genes for each cell subpopulation; dot size represents the proportion of expressing cells, and color represents the average expression level.

Principal component analysis (PCA) was performed on the integrated data, and the first 30 principal components were selected based on the ElbowPlot for UMAP dimensionality reduction and clustering (resolution = 0.8). Cell subpopulations were annotated based on canonical marker genes (e.g., Ms4a3 for neutrophils, Epcam for epithelial cells, Pecam1 for endothelial cells, Col1a1 for fibroblasts, Cd3d for T cells, Ms4a1 for B cells, Csf1r for macrophages), combined with comprehensive annotation using the heatmap of the top 10 marker genes for each cluster shown in Figure 3C.

#### 2.5.3 Differential expression analysis and functional enrichment

Differentially expressed genes (DEGs) between the control (Sham) and sepsis (Septiclung) groups were identified using the FindMarkers function (parameters: logFC > 0.25, p_val_adj < 0.05). KEGG pathway enrichment analysis and GSEA (Gene Set Enrichment Analysis, using MSigDB hallmark gene sets) were performed on the DEGs using the clusterProfiler R package to reveal potential biological functional alterations. Upregulated and downregulated genes were subjected to enrichment analysis separately, and the results were displayed as bubble plots and bar charts.

#### 2.5.4 Pseudotime analysis

Pseudotime analysis of alveolar macrophages was performed using the Monocle 3 R package. Cell trajectories were first constructed via learn_graph, and root cells were then selected based on cell states and known marker genes (e.g., cells with high Gm47719 expression). Pseudotime was calculated, and dynamic changes in gene expression along pseudotime were analyzed.

#### 2.5.5 Cell communication analysis

The CellChat R package (using the default ligand–receptor database) was applied to infer ligand–receptor interaction networks among cells. Differences in signaling pathway strength between the Sham and Sepsis groups were compared, focusing on the altered communication of alveolar macrophages as signal senders with other cell types. Key interaction pairs were visualized using heatmaps and chord diagrams.

### 2.6 HE staining

Following the carbon dioxide euthanasia of the experimental mice, pathological sections were prepared, lung tissue specimens were examined under an optical microscope for pulmonary edema, hemorrhage, and alveolar inflammation, and electronic photographs were taken. The steps involved in preparing pathological sections were as follows: (1) lung tissue specimens were removed from 4% paraformaldehyde; (2) dehydration: specimens were placed in increasing concentrations of alcohol (70–100%) for 30 min each; (3) clearing: tissue blocks were placed in xylene I and xylene II for 10 min each until transparent, replacing alcohol in the tissue blocks; (4) wax immersion and embedding cassettes, embedded in liquid paraffin, and allowed to cool and solidify at room temperature; (5) sectioning and mounting; and (65 °C oven. After 10 min of hematoxylin staining, the sections were washed with distilled water, differentiated in 1% alcohol for 10 s, and then rinsed again with distilled water. After three minutes of eosin counterstaining, the sections were dehydrated in increasing alcohol concentrations (70, 95, and 100%) for two minutes each, and then they were cleaned in xylene. Neutral resin was used to attach the sections, which were then coverslipped.

### 2.7 ELISA

Serum levels of MDA, SOD, GPX, IL-1β, IL-18, and GSDMD were measured. (1) Specimens were removed from the −80 °C freezer and thawed at room temperature for 2 h, centrifuged at 1000 x g for 15 min, and set aside. (2) Standard working solutions were prepared according to the kit instructions and added to the first two columns of the plate, with two wells per concentration as duplicates, 100 μL per well. Test samples were added to other wells, 100 μL per well. The plate was covered and incubated at 37 °C for 120 min. (3) Liquid was discarded, and the plate was blotted dry. Biotin-labeled antibody working solution was diluted, and 100 μL was added to each well, mixed, and incubated at 37 °C for 1 h. (4) Wash buffer was prepared according to the kit instructions. After discarding the liquid, the plate was washed three times with 350 μL wash buffer per well, soaked for 1 min each time, and blotted dry. (5) HRP-labeled avidin working solution was diluted according to the kit instructions, and 100 μL was added to each well, covered, and incubated at 37 °C for 60 min. (6) Liquid was discarded, and the plate was washed five times as in step 3. (7) Substrate solution (90 μL) was added to each well, covered, and incubated at 37 °C in the dark for 15 min. (8) Stop solution (50 μL) was added to each well to terminate the reaction. (9) Optical density was measured at 450 nm using a microplate reader within 5 min of stopping the reaction. The concentration of cytokines was calculated based on the standard curve generated by the standard working solutions.

### 2.8 Western blot

Total protein was extracted from lung tissue. Lung tissue stored in liquid nitrogen was minced in an EP tube, and 1 mL of red blood cell lysis buffer was added. The tissue was homogenized and centrifuged at 4 °C at 3500 rpm to obtain the pellet. The supernatant was discarded, and lysis buffer RIPA and protease inhibitors were added. The sample was incubated on ice for 30 min, vortexed every 10 min, and protein loading buffer was added. The sample was heated at 100 °C for 10 min and stored at −20 °C. Western blot was performed using a kit for gel preparation, transfer, primary and secondary antibody incubation, and ECL development. Band intensity was analyzed using ImageJ software. The primary antibodies used in this study included anti-Siglec-5 (PA5-11675, Thermo), anti-SIRPα (ab191419, abcam), anti-HSF1 (ab242138, abcam), anti-phospho-SYK (2,710 T, CST), anti-SYK (13,198 T, CST), anti-phospho-ERK (44-680G, Thermo), anti-ERK (MA5-15134, Thermo), anti-phospho-ELK1 (PA5-104832, Thermo), anti-ELK1 (MA5-32109, Thermo), anti-SHP2 (ab300579, abcam), anti-phospho-SHP2 (44-554G, Thermo), and anti-NOX4 (ab319055, abcam), anti-NLRP3 (ab263899, abcam), anti-IL-1β (ab234437, abcam), anti-GSDMD (ab219800, abcam). GAPDH (ab181602, abcam) was used as a loading control. All primary antibodies were diluted at 1:1000.

### 2.9 Co-immunoprecipitation (co-IP) assays

To investigate the potential protein–protein interactions between Siglec-5 and HSF1, as well as ELK1 and SIRPα, co-immunoprecipitation (Co-IP) assays were performed using RAW264.7 cell lysates. Approximately 1 × 10^7^ cells were harvested and lysed on ice for 30 min in pre-chilled modified RIPA lysis buffer (50 mM Tris–HCl, pH 7.4, 150 mM NaCl, 1% NP-40, 0.25% sodium deoxycholate, 1 mM EDTA) supplemented with freshly added protease inhibitor cocktail and 1 mM PMSF. After centrifugation at 12,000 × g for 15 min at 4 °C, the supernatants were collected, and protein concentrations were determined using a BCA assay kit. For each immunoprecipitation, 500 μg of total protein lysate was incubated with 1 μg of anti-Siglec-5 (PA5-11675, Thermo) or anti-ELK1 (MA5-32109, Thermo), or control rabbit IgG (Abcam) antibodies overnight at 4 °C with gentle rotation. Subsequently, 20 μL of pre-washed Protein A/G magnetic beads (Invitrogen) were added to each sample and incubated for an additional 2 h at 4 °C. The bead–antibody–antigen complexes were magnetically separated and washed five times with ice-cold lysis buffer to remove non-specific binding. After the final wash, the immunoprecipitated complexes were eluted by resuspending the beads in 2 × SDS-PAGE loading buffer and boiling at 95 °C for 10 min. The eluted proteins were then subjected to Western blot analysis, where the presence of co-precipitated HSF1 (ab242138, Abcam) or SIRPα (ab191419, Abcam) was detected using specific primary antibodies. To avoid interference from denatured IP antibody heavy chains, HRP-conjugated light-chain-specific secondary antibodies (e.g., Jackson ImmunoResearch, 211-032-171) were used for all rabbit primary antibodies during blotting. Input controls representing 5% of the initial lysate were included to confirm the expression of target proteins.

### 2.10 *In vitro* cell experiments

RAW264.7 cells were cultured in DMEM medium supplemented with 10% heat-inactivated fetal bovine serum and 5% penicillin–streptomycin in a 37 °C, 5% CO2 incubator. Cells were divided into eight groups: NC group, LPS group, Siglec5-OE group, Siglec5-OE + Rocaglamide group, Siglec5-OE + Rocaglamide+Piceatannol group, Siglec5-OE + Rocaglamide+Piceatannol+mSIRK group, Siglec5-OE + Rocaglamide+Piceatannol+mSIRK+TDE group, and Siglec5-OE + Rocaglamide+Piceatannol+mSIRK+TDE + SIRPα-OE group. The NC group received no treatment. The LPS group was treated with 100 ng/mL LPS. The HSF1 inhibitor Rocaglamide (100 nM), SYK inhibitor Piceatannol (10 μM), ERK1/2 activator mSIRK (10 μM), and ELK1 inhibitor TAT-DEF-Elk-1 (TDE, 10 μM) were used to treat the respective groups. For lentiviral transduction, Siglec5-OE (constructed using the mouse Siglec-5 coding sequence, Gene ID: 233186) and SIRPα-OE lentiviruses (both at a titer of 1 × 10^8^ TU/mL, MOI = 10) were added to the corresponding groups according to the manufacturer’s instructions. The specificity of the anti-Siglec-5 antibody (MA5-48652, Thermo) for mouse Siglec-5 has been validated by the manufacturer and confirmed by our overexpression efficiency data. Transduction efficiency was verified by RT-qPCR 48 h post-transfection, showing >85% efficiency (Siglec-5 and SIRPα mRNA increased >6-fold and >5-fold, respectively). All experiments were performed with at least three independent biological replicates (*n* = 3).

### 2.11 Isolation, culture, and experimental treatment of mouse primary macrophages

Femurs and tibiae were obtained from 6- to 8-week-old C57BL/6 J mice. The mice were euthanized by cervical dislocation and immersed in 75% ethanol for 5 min for disinfection. Bone marrow cavities were flushed with PBS containing 1% penicillin–streptomycin. The cell suspension was filtered through a 70 μm cell strainer and centrifuged. Red blood cells were removed using red blood cell lysis buffer. The remaining cells were resuspended in complete medium (DMEM supplemented with 10% FBS and 1% penicillin–streptomycin) containing 20 ng/mL M-CSF and cultured at 37 °C in 5% CO₂ for 7 days (medium was changed every 2 days). The purity of F4/80^+^ cells reached ≥90% before being used for experiments.

Mature primary macrophages were seeded into 6-well plates at a density of 1 × 10^6^ cells/well. After adherence, cells were treated according to the same grouping scheme as used for RAW264.7 cells: NC group (no treatment), LPS group (stimulation with 100 ng/mL LPS for 24 h), Siglec5-OE group (lentiviral infection for 48 h followed by culture for 24 h), and various combination treatment groups. All groups were incubated at 37 °C in 5% CO₂ for the indicated times, after which samples were collected for analysis.

### 2.12 Flow cytometry for apoptosis rate

Cells from each group were collected into centrifuge tubes, centrifuged at 2000 rpm for 5 min at room temperature, and resuspended in 4 °C pre-cooled PBS. After centrifugation, the supernatant was discarded. Cells were resuspended in 1 × Annexin-binding buffer at a concentration of 1 × 10^6/mL. A 100 μL cell suspension was transferred to a 5 mL flow tube, and 5 μL eGFP Annexin V and 2 μL PI were added, incubated for 15 min, and 400 μL buffer was added. The apoptosis rate was quantified as the percentage of Annexin V-positive cells using FlowJo software.

### 2.13 Confocal fluorescence localization for ROS fluorescence expression

To enable the probe to completely interact with the cells, cells from each group were collected, resuspended in 0.01 mol/L PBS and diluted DCFH-DA, and incubated at 37 °C for an hour. The cell pellet was resuspended in PBS after the single-cell suspension was centrifuged for ten minutes at 1000 × g. A spectrum laser confocal microscope was used to detect fluorescence, and ImageJ software was used to quantify the data.

### 2.14 Immunofluorescence staining

Following antigen extraction, tissue autofluorescence was quenched, serum blocking, primary and secondary antibody incubation, DAPI nuclear staining, and mounting with anti-fade mounting media. Pictures were taken, with the CY3 channel displaying red positive signals and the DAPI channel displaying blue nuclei. Immunofluorescence pictures were quantified using ImageJ software using the formula mean optical density (MOD) = integrated optical density (IOD)/area of target protein distribution.

### 2.15 Statistical analysis

GraphPad Prism 8.0.2 and SPSS 26.0 were used to analyze the data. The mean ± SD of at least three separate experiments is used to display the results. Tukey’s HSD post-hoc test was employed after one-way ANOVA to control the family-wise error rate for multi-group comparisons. Pilot experiments and prior research were used to estimate sample sizes (*n* = 3–7 per group); no formal power calculation was carried out. Mice were divided into experimental groups at random, and while evaluating the results, researchers were blind to the group assignments. After adjustment, a two-sided *p* < 0.05 was deemed statistically significant.

## 3 Results

### 3.1 Bioinformatics screening and experimental validation of key signaling pathways

Boxplots and PCA were used to corroborate data preparation and normalization, demonstrating how well the ComBat method eliminated batch effects. 1,000 substantially dysregulated genes were found in the peripheral blood sample (GSE28750) and 142 in the lung tissue dataset (GSE10361) using differential expression analysis. 197 key overlapping genes were found by intersection analysis using Venn diagram visualization. These genes were then submitted to functional enrichment analysis. According to Gene Ontology (GO) analysis, these genes were highly enriched in biological processes involving NLRP3 inflammasome assembly and the response to bacterial molecules; molecular functions associated with protein tyrosine kinase activity and Toll-like receptor (TLR) binding; and cellular components such as neutrophil-specific granules and inflammasome complexes. The enrichment of these genes in the NF-kappa B signaling pathway, neutrophil extracellular trap formation, and the NOD-like receptor signaling pathway was further revealed by Kyoto Encyclopedia of Genes and Genomes (KEGG) pathway analysis, indicating their possible role in the systemic immune response and local tissue damage linked to sepsis.

Potential molecular connections were revealed by correlation network analysis. Significant positive correlations between certain molecular nodes were shown using Spearman correlation matrices, indicating coordinated regulation processes. For example, co-expression of the C-type lectin receptor Dectin and damage-associated molecular patterns (DAMPs) suggests a possible synergistic function in pathogen identification. Additionally, the correlations between SYK, SHP2, and ERK1/2, as well as the linkage between Dectin and spleen tyrosine kinase (SYK), suggest that the SYK-SHP2-ERK signaling axis may be involved in inflammatory signal transduction. Lastly, the hypothesis that the NLRP3 inflammasome may contribute to the inflammatory cascade and programmed cell death in sepsis-induced lung injury is supported by the strong correlations found between NLRP3 and important inflammatory markers IL − 1β, IL − 18, IL − 6, as well as pyroptosis-related proteins (GSDMD, caspase-1/4).

**Figure 4:**
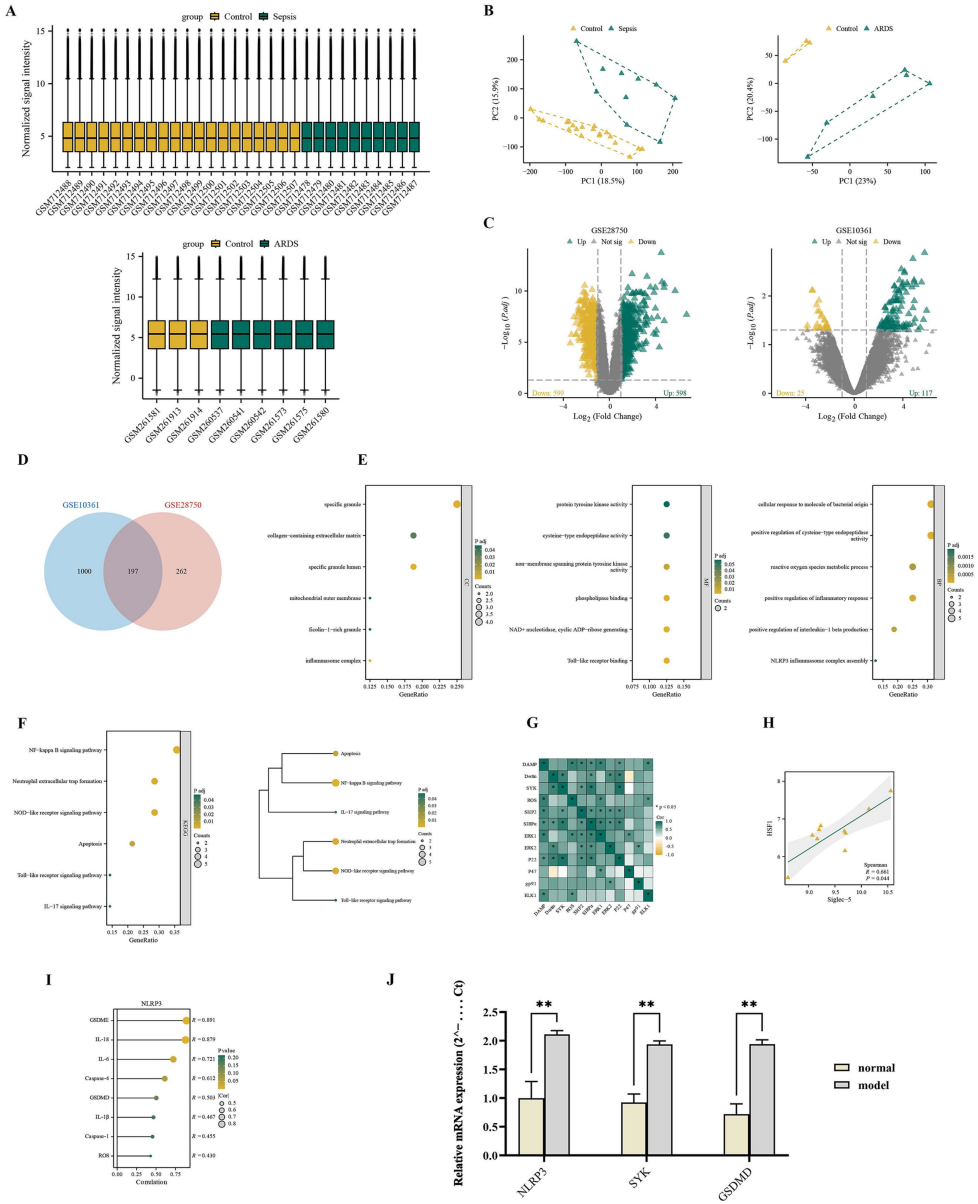
**(A)** Boxplots for quality assessment of standardized datasets GSE28750 and GSE10361; **(B)** PCA plots for batch effect correction of datasets GSE28750 and GSE10361; **(C)** Volcano plots for differential expression gene distribution in datasets GSE28750 and GSE10361; **(D)** Venn diagram for intersection analysis of differentially expressed genes in datasets GSE28750 and GSE10361; **(E)** (E1) GO enrichment - cellular component bubble plot; (E2) GO enrichment - molecular function bubble plot; (E3) GO enrichment - biological process bubble plot; **(F)** (F1) KEGG enrichment analysis bubble plot; (F2) KEGG pathway functional clustering plot; **(G)** Heatmap of key molecular correlation network; **(H)** Scatter plot of Siglec-5 and HSF1 correlation; **(I)** Lollipop plot for multi-dimensional regulatory relationship analysis of NLRP3 inflammasome.

We used qRT-PCR to measure the transcriptional levels of the selected main targets in our experimental model in order to confirm the bioinformatics predictions made from the public datasets. The mRNA expression levels of NLRP3, SYK, and GSDMD were considerably higher in the experimental group than in the control group (all *p* < 0.001), which is consistent with the in silico study. Furthermore, there was a statistically significant increase (*p* < 0.01) in the expression of MAPK3 (ERK1). These experimental findings validate the major molecular drivers found by our comprehensive bioinformatics analysis by confirming the activation of the NLRP3 inflammasome and SYK-ERK signaling pathways (Figure 4).

### 3.2 Construction of the lung single-cell atlas and identification of cell subpopulations

Through unsupervised clustering analysis, we identified seven major cell types in mouse lung tissue, including alveolar macrophages, B cells, CD4^+^ T cells, fibroblasts, lung endothelial cells, lung epithelial cells, and neutrophils. UMAP visualization showed that different cell subpopulations were clearly demarcated in two-dimensional space (Figure 3A). Comparison of cell distributions between the Sham (control) and Septiclung (sepsis) groups revealed a marked alteration in the cellular subpopulation structure in the Septiclung group (Figure 3B). To precisely identify each cell subpopulation, we presented the top 10 specifically highly expressed marker genes for each cluster (Figures 3C,D). Lung epithelial cells specifically highly expressed genes such as 1600002D24Rik, Gm50255, Il23a, 4931406B18Rik, Gm19510, Gm36079, Fu2, Gm1647, Gm47719, and Ofcc1; lung endothelial cells specifically highly expressed Gpr156, Ctsp., Lad1, Kcnj15, C1ql2, 4931431B13Rik, Smoc1, Igf2bp1, Egflam1, and Slc1a2; alveolar macrophages specifically highly expressed Gm35330, It25, Gm15056, Fscn1, Ostam, Gm42601, Cd163, Spic, Il12b, and Tm4sf5; neutrophils specifically highly expressed 4930463O16Rik, Gm8439, Ltf, Ngp, Ldhc, C230013L11Rik, Garem1, Camp, Thbs1, and Ighg2i; CD4^+^ T cells specifically highly expressed Gm2682, 2310026D22Rik, Gm37469, Ilk, Izumo1r, 5830468F06Rik, Gm26112, Klra10, Klra9, and 5830411N06Rik; B cells specifically highly expressed Igkap3, Pax5, 2010309G21Rik, Ighg2b, Srp1, Esco2, Gm12158, Hist1h2af, Ighd, and Gm43388; fibroblasts specifically highly expressed Entpd2, Nox4, Fendrr, Pck4, Mmp3, Kank4, Tspan18, Scara3, Pdgfd, and Serpinb3m.

### 3.3 Transcriptomic alterations and functional enrichment in septic lung tissue

**Figure 5:**
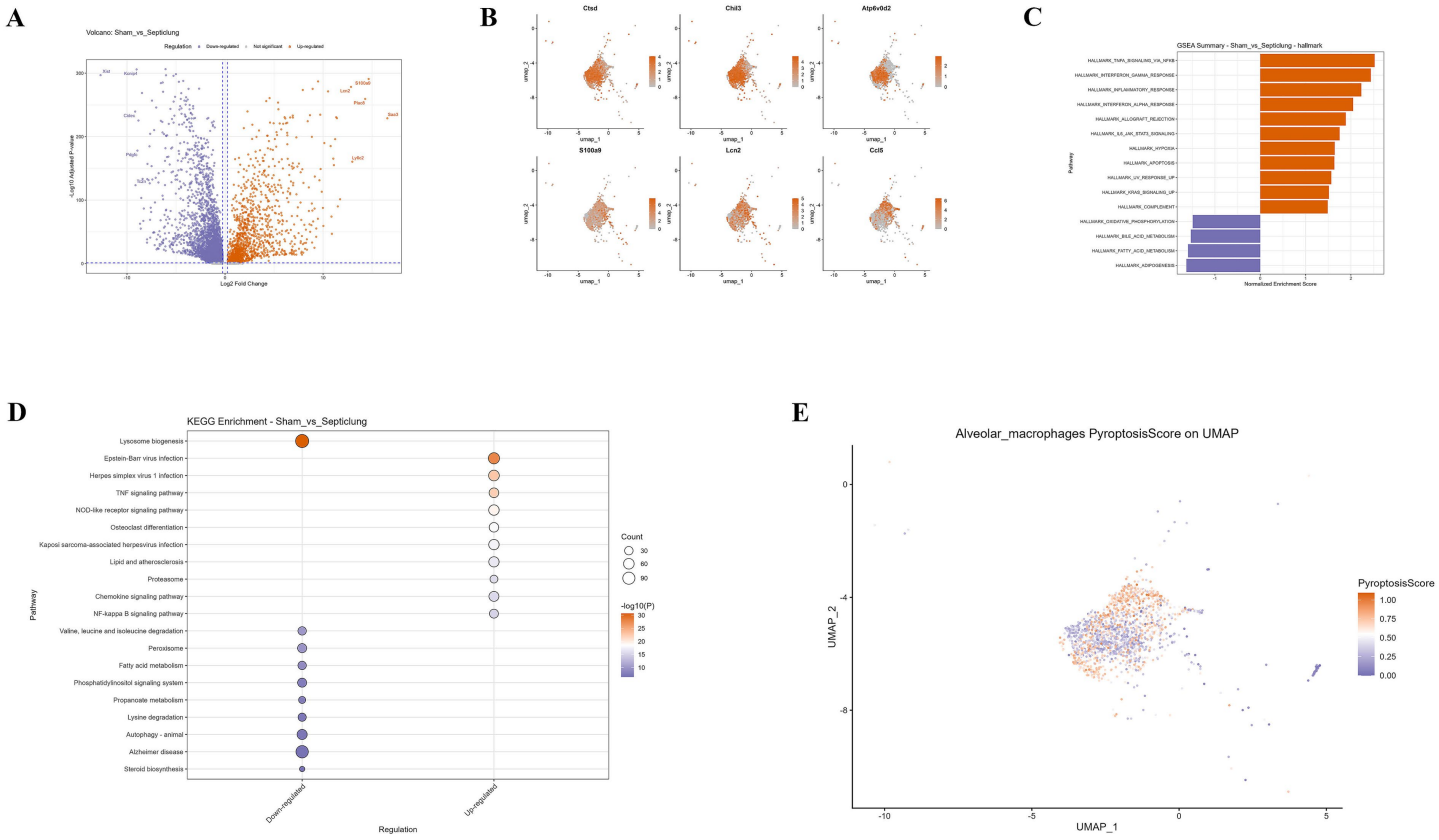
Transcriptomic alterations and functional enrichment in septic lung tissue. **(A)** Volcano plot of differentially expressed genes between the Sham and Sepsis groups. **(B)** Violin plots showing the expression levels of key inflammatory genes in each cell subpopulation. **(C)** Gene Set Enrichment Analysis (GSEA) showing the activation of inflammation-related pathways. **(D)** Bubble plot of KEGG pathway enrichment analysis of differentially expressed genes. **(E)** Distribution of the cell score based on pyroptosis-related gene sets (PyroptosisScore) projected onto the UMAP plot.

To explore the molecular changes induced by sepsis, we compared gene expression differences between the Septiclung and Sham groups. The volcano plot revealed a large number of significantly altered genes (Figure 5A). Among them, significantly upregulated genes (orange dots) mainly included inflammation- and neutrophil activation-related genes such as S100a9, Lcn2, Plac8, Saa3, and Ly6c2. Significantly downregulated genes (purple dots) included Xist, Kcnip4, Cidec, Pdgfc, and Tsc22d1.

Further analysis of the number of differentially expressed genes in each major cell type showed that neutrophils, alveolar macrophages, and monocytes harbored the largest numbers of DEGs, indicating that these immune cells underwent profound transcriptional reprogramming during septic lung injury (Figure 5B). KEGG pathway enrichment analysis revealed that upregulated genes were significantly enriched in inflammation-related pathways such as “Cytokine–cytokine receptor interaction”, “NF-kappa B signaling pathway”, and “TNF signaling pathway”, whereas downregulated genes were enriched in metabolism-related pathways such as “Fatty acid metabolism” and “Peroxisome” (Figure 5D). GSEA also confirmed that gene sets associated with inflammatory response and immune response were significantly enriched in the Septiclung group (Figure 5C).

In addition, we calculated the pyroptosis score of alveolar macrophages. The results showed that the pyroptosis score of alveolar macrophages in the Septiclung group was significantly higher than that in the Sham group (Figure 5E), suggesting that alveolar macrophage pyroptosis may play a crucial role in septic lung injury. These transcriptomic observations provided a rationale for subsequent computational prediction and experimental interrogation of candidate regulatory molecules such as Siglec-5.

### 3.4 Dynamic evolutionary trajectory of alveolar macrophages and in silico prediction of Siglec-5 function

**Figure 6:**
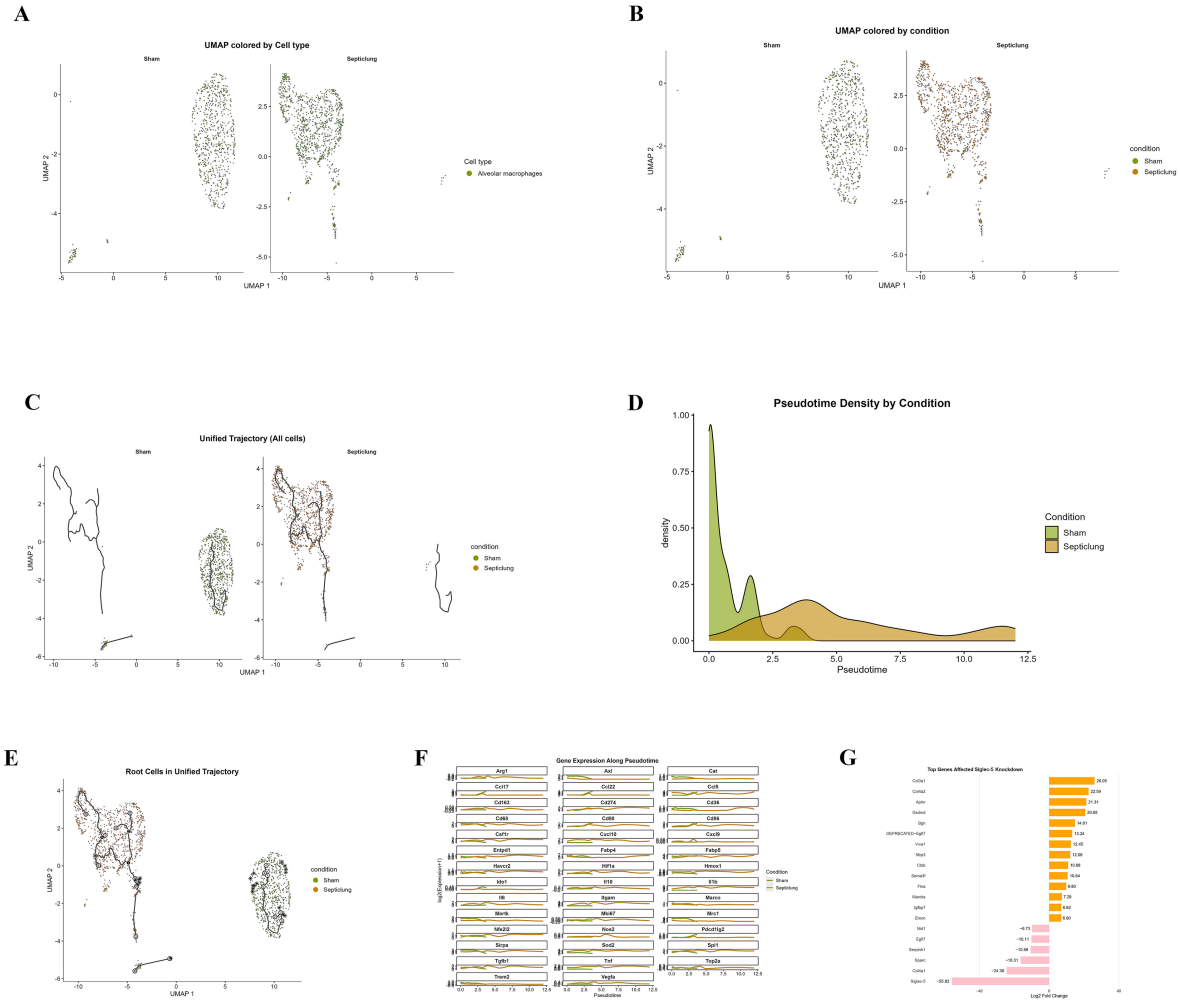
Pseudotime trajectory evolution of alveolar macrophages in sepsis and in silico Siglec-5 perturbation analysis. **(A)** UMAP visualization of alveolar macrophages colored by cell type. **(B)** UMAP plot colored by experimental condition (Sham vs. Septiclung). **(C)** Pseudotime trajectory reconstruction showing the evolutionary path of macrophages from the Sham state (early) to the Septiclung state (late). **(D)** Pseudotime density distribution. The plot shows that Septiclung cells are significantly enriched in the late phase of the trajectory compared to Sham cells. **(E)** Identification of root cells (starting point) used to define the directionality of the pseudotime trajectory. **(F)** Dynamic expression trends of key genes along pseudotime. Pro-inflammatory genes (Cd86, Il1b, Nos2, Tnf) are upregulated, while homeostatic genes (Arg1, Mrc1) are downregulated as pseudotime progresses. **(G)** In silico virtual knockout analysis of Siglec-5 using the scFAN algorithm. The bar chart displays predicted differentially expressed genes, showing upregulation of pro-inflammatory markers (Nlrp3) and downregulation of matrix homeostasis genes (Col4a1, Sparc, Serpinh1) following simulated Siglec-5 loss.

To dissect the pathological evolution of alveolar macrophages in sepsis and its underlying molecular mechanisms, we performed a systematic study combining pseudotime analysis with an in silico (virtual) Siglec-5 perturbation analysis (Figure 6). The virtual knockout analysis was designed as a hypothesis-generating tool rather than a mechanistic validation.

First, UMAP dimensionality reduction visualized the distinct distribution of alveolar macrophages across different conditions (Figures 6A,B). We then reconstructed a continuous pseudotime trajectory to recapitulate the transition of these macrophages from a healthy to a diseased state (Figure 6C). Sepsis induced a rapid pathological shift: cells from the Sham group were predominantly located at the early phase (root) of the trajectory, while cells from the Septiclung group were markedly enriched in the late phase (Figures 6C,D). Notably, the trajectory exhibited a clear branching pattern, suggesting that alveolar macrophages undergo fate decisions and differentiate into distinct functional subpopulations as the disease progresses. We further identified the root cells to define the directionality of this evolution (Figure 6E). Gene expression dynamics analysis based on the defined trajectory direction revealed that pro-inflammatory genes (e.g., Cd86, Il1b, Nos2, Tnf) were significantly upregulated as pseudotime progressed, while homeostatic genes (e.g., Arg1, Mrc1) were downregulated (Figure 6F), indicating a progressive polarization of macrophages toward a pro-inflammatory phenotype.

To predict the potential consequences of Siglec-5 loss, we performed a virtual gene knockout analysis using the scFAN algorithm on the alveolar macrophage gene expression matrix. This computational simulation is intended for hypothesis generation only, does not represent actual genetic deletion, and its predictions are subject to limitations of network inference and require experimental validation.

The virtual knockout analysis predicted that the loss of Siglec-5 would lead to significant reprogramming of downstream gene expression patterns, with predicted changes converging on oxidative stress, apoptosis, and NLRP3 inflammasome activation (Figure 6G): NLRP3 inflammasome and pro-inflammatory signal upregulation: Nlrp3 gene expression was predicted to be significantly upregulated (Log2 Fold Change > 0) after Siglec-5 knockdown. The predicted upregulation of NLRP3, a key component of the inflammasome, suggested that Siglec-5 loss might release the inhibition of inflammasome assembly, potentially aggravating Caspase-1 activation and downstream pro-inflammatory cytokine release.

Oxidative stress and apoptosis-related matrix remodeling: The analysis also predicted that genes linked to antioxidant defense and extracellular matrix integrity, namely Col4a1, Sparc, and Serpinh1, would be markedly downregulated (Figure 6G). These predicted changes implied that the loss of Siglec-5 could compromise both alveolar structural integrity and cellular defense against oxidative stress, potentially contributing to tissue damage and apoptosis.

These computational predictions provided a testable hypothesis—that Siglec-5 exerts a protective role by suppressing NLRP3-driven inflammation and oxidative damage—which directly motivated the subsequent *in vivo* and *in vitro* loss- and gain-of-function experiments described in the following sections.

### 3.5 Intercellular communication network remodeling

**Figure 7:**
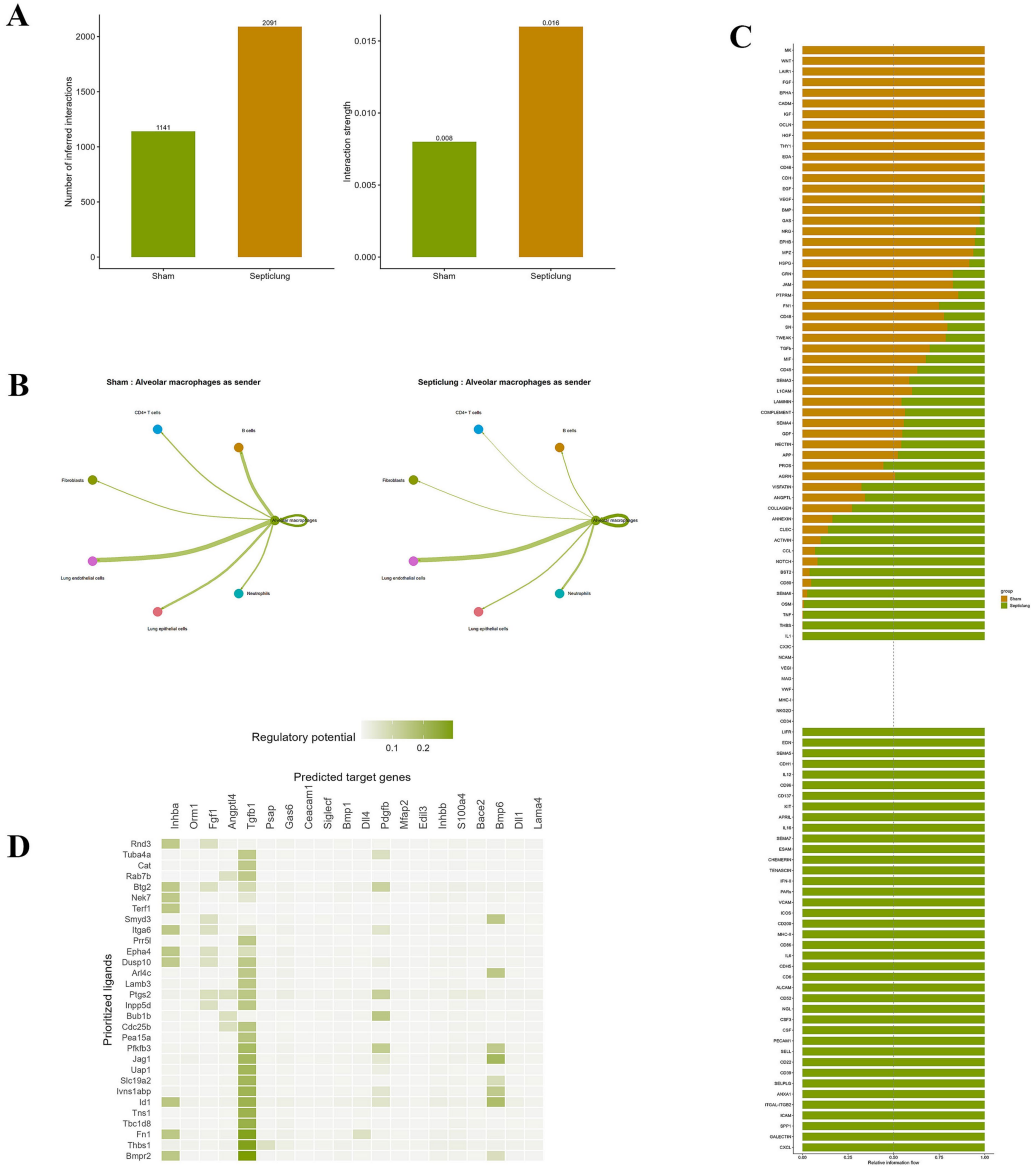
Remodeling of the intercellular communication network. **(A)** Intercellular interaction network diagram, where node size represents cell number and edge thickness represents interaction strength. **(B)** Communication network diagram centered on alveolar macrophages. **(C)** Heatmap of the strength of intercellular ligand–receptor interactions. **(D)** Heatmap of ligand–target gene regulatory potential.

Cell communication analysis revealed that the Septiclung group had much more intercellular connections than the Sham group, both in terms of quantity and intensity (Figure 7A). Alveolar macrophage contacts with neutrophils, endothelial cells, fibroblasts, and other cells were significantly increased in the Septiclung group, according to network analysis using alveolar macrophages as signal senders (Figure 7B). Stronger communication signals between many cell pairs in the Septiclung group were shown by the heatmap, which showed the intensity of intercellular connections (Figure 7C). Lastly, important signaling pathways were discovered by ligand–target gene regulatory potential analysis. The heatmap revealed that the regulatory potential of ligands like Tnfsf12, Tnfsf13, Il1b, Tnf, and Ccl2 on target genes (including Nfkb1, Stat1, Jun, and Fos) was markedly increased in the Septiclung group (Figure 7D). This suggests that in septic environments, alveolar macrophages release a variety of inflammatory molecules that strongly trigger downstream inflammatory signaling pathways in other cells, accelerating the development of lung damage.

### 3.6 Siglec-5 inhibits LPS-induced lung injury

To further validate the protective role of Siglec-5 observed above, we investigated the effects of Siglec-5 knockdown on LPS-induced lung injury. The successful overexpression and knockdown efficiency of Siglec-5 were confirmed by qRT-PCR (Figure S1). Compared to the sham group, the Model group exhibited severe lung tissue damage (Figure 1A), increased levels of MDA, IL-1β, IL-18, and GSDMD, decreased levels of SOD and GPX (Figures 1B,C), elevated protein expression of NOX4, SIRPα, and NLRP3, and decreased protein expression of Siglec-5 and HSF1 (Figure 1D); compared to the Model group, the Siglec5-OE group showed improved lung injury, reduced levels of MDA, IL-1β, IL-18, and GSDMD, increased levels of SOD and GPX, decreased protein expression of NOX4, SIRPα, and NLRP3, and increased protein expression of Siglec-5 and HSF1; compared to the Siglec5-OE group, the Siglec5-OE + SIRPα-OE group exhibited significantly more severe lung tissue damage, increased levels of MDA, IL-1β, IL-18, and GSDMD, decreased levels of SOD and GPX, significantly elevated protein expression of NOX4, SIRPα, and NLRP3, and decreased protein expression of Siglec-5 and HSF1.

To further validate the protective role of Siglec-5 observed above, we investigated the effects of Siglec-5 knockdown on LPS-induced lung injury. Compared to the sham group, the model group exhibited severe lung tissue damage (Figure 2A), increased levels of MDA, IL-1β, IL-18, and GSDMD, and decreased levels of SOD and GPX (Figures 2B,C); compared to the model group, the siglec5-KD group showed significantly more severe lung tissue damage, further increased levels of MDA, IL-1β, IL-18, and GSDMD, and significantly decreased levels of SOD and GPX.

### 3.7 Siglec-5 induces through the HSF1/SYK/ERK1/2/SIRPα signaling pathway

**Figure 8:**
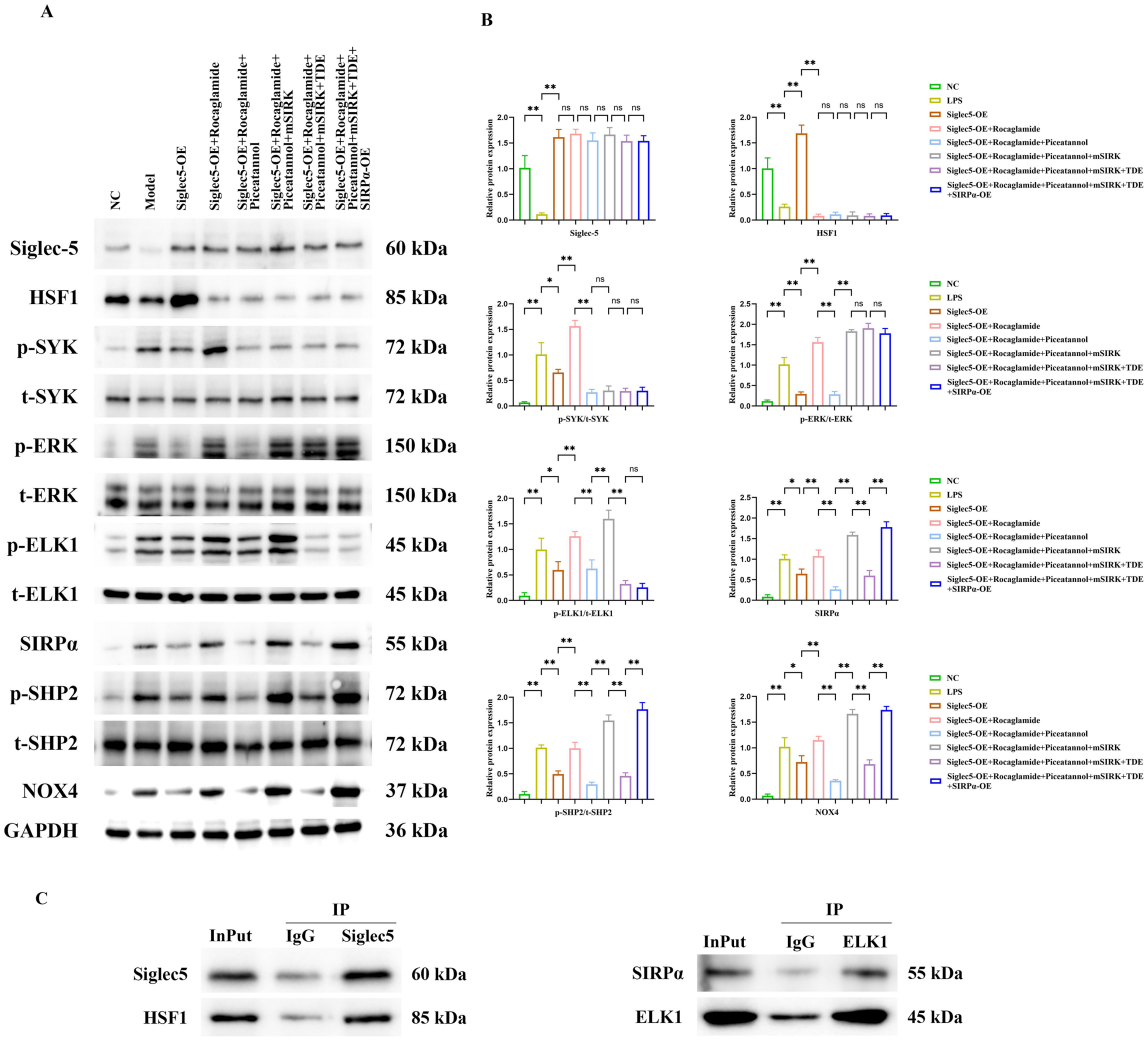
Siglec-5 induces through the HSF1/SYK/ERK1/2/SIRPα signaling pathway. (**A)** Western blot for detecting Siglec5, HSF1, NLRP3, p-SYK, p-ERK, p-ELK1, SIRPα, p-SHP2, and NOX4 protein expression. **(B)** Data statistics. Relative protein expression was quantified by densitometry using ImageJ and normalized to corresponding internal reference proteins (e.g., GAPDH or total protein). Data represent mean ± SD from three independent experiments. **(C)** CO-IP experiments confirmed the interaction; **(C)** Co-IP assays verified the interactions between Siglec5/HSF1 and ELK1/SIRPa.

Compared to the NC group, the LPS group showed decreased protein expression of Siglec5 and HSF1, and increased protein expression of p-SYK, p-ERK, p-ELK1, SIRPα, p-SHP2, and NOX4; compared to the LPS group, the Siglec5-OE group showed increased protein expression of Siglec5 and HSF1, and decreased protein expression of p-SYK, p-ERK, p-ELK1, SIRPα, p-SHP2, and NOX4; compared to the Siglec5-OE group, the Siglec5-OE + Rocaglamide group showed no significant difference in Siglec5 protein expression, decreased HSF1 protein expression, and significantly increased protein expression of p-SYK, p-ERK, p-ELK1, SIRPα, p-SHP2, and NOX4; compared to the Siglec5-OE + Rocaglamide group, the Siglec5-OE + Rocaglamide+Piceatannol group showed no significant difference in Siglec5 and HSF1 protein expression, and decreased protein expression of p-SYK, p-ERK, p-ELK1, SIRPα, p-SHP2, and NOX4; compared to the Siglec5-OE + Rocaglamide+Piceatannol group, the Siglec5-OE + Rocaglamide+Piceatannol+mSIRK group showed no significant difference in Siglec5, HSF1, and p-SYK protein expression, and increased protein expression of p-ERK, p-ELK1, SIRPα, p-SHP2, and NOX4; compared to the Siglec5-OE + Rocaglamide+Piceatannol+mSIRK group, the Siglec5-OE + Rocaglamide+Piceatannol+mSIRK+TDE group showed no significant difference in Siglec5, HSF1, p-SYK, and p-ERK protein expression, and decreased protein expression of p-ELK1, SIRPα, p-SHP2, and NOX4; compared to the Siglec5-OE + Rocaglamide+Piceatannol+mSIRK+TDE group, the Siglec5-OE + Rocaglamide+Piceatannol+mSIRK+TDE + SIRPα-OE group showed no significant difference in Siglec5, HSF1, p-SYK, p-ERK, and p-ELK1 protein expression, and significantly increased protein expression of SIRPα, p-SHP2, and NOX4 (Figure 8).

To confirm protein interactions, Co-IP analysis was performed. Using an anti-Siglec5 antibody, HSF1 was specifically co-precipitated, while anti-ELK1 antibody captured SIRPα. Input lysates verified protein expression. These results indicate a protein association between Siglec5 and HSF1, and between ELK1 and SIRPα, supporting the proposed signaling axis in Figure 8C.

### 3.8 Siglec-5 inhibits LPS-induced lung injury by suppressing ROS and pyroptosis

**Figure 9:**
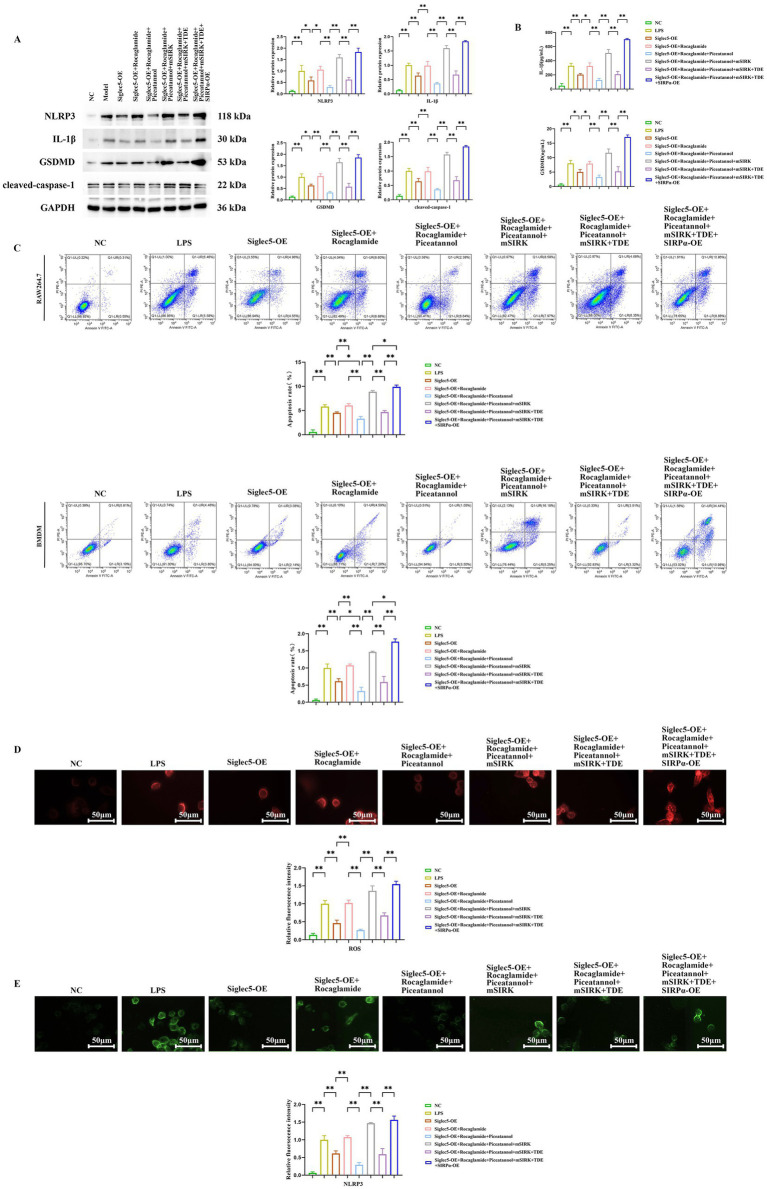
Siglec-5 inhibits LPS-induced lung injury by suppressing ROS and pyroptosis. **(A)** Western blot for detecting NLRP3, IL-1β, and GSDMD protein expression. Densitometric quantification was performed using ImageJ software. **(B)** ELISA for measuring IL-1β and GSDMD levels. **(C)** Flow cytometry analysis of apoptosis in RAW264.7 cells and primary bone marrow-derived macrophages (BMDMs). The apoptosis rate is presented as the percentage of positive cells. **(D)** Fluorescent probe for detecting ROS fluorescence intensity. Fluorescence intensity was measured using ImageJ software. **(E)** Immunofluorescence for detecting NLRP3 fluorescence intensity. Mean fluorescence intensity was analyzed using ImageJ.

Compared to the NC group, the LPS group showed increased protein expression of NLRP3, IL-1β, and GSDMD, elevated levels of IL-1β and GSDMD, increased cell apoptosis, and enhanced ROS and NLRP3 fluorescence intensity. Similar anti-apoptotic effects of Siglec5 overexpression were confirmed in mouse bone marrow-derived macrophages (BMDMs), where Siglec5-OE significantly reduced LPS-induced apoptosis (Figure 9C). Compared to the LPS group, the Siglec5-OE group showed decreased protein expression of NLRP3, IL-1β, and GSDMD, reduced levels of IL-1β and GSDMD, decreased cell apoptosis, and weakened ROS and NLRP3 fluorescence intensity; compared to the Siglec5-OE group, the Siglec5-OE + Rocaglamide group showed increased protein expression of NLRP3, IL-1β, and GSDMD, elevated levels of IL-1β and GSDMD, increased cell apoptosis, and enhanced ROS and NLRP3 fluorescence intensity; compared to the Siglec5-OE + Rocaglamide group, the Siglec5-OE + Rocaglamide+Piceatannol group showed decreased protein expression of NLRP3, IL-1β, and GSDMD, reduced levels of IL-1β and GSDMD, decreased cell apoptosis, and weakened ROS and NLRP3 fluorescence intensity; compared to the Siglec5-OE + Rocaglamide+Piceatannol group, the Siglec5-OE + Rocaglamide+Piceatannol+mSIRK group showed significantly increased protein expression of NLRP3, IL-1β, and GSDMD, elevated levels of IL-1β and GSDMD, significantly increased cell apoptosis, and enhanced ROS and NLRP3 fluorescence intensity; compared to the Siglec5-OE + Rocaglamide+Piceatannol+mSIRK group, the Siglec5-OE + Rocaglamide+Piceatannol+mSIRK+TDE group showed significantly decreased protein expression of NLRP3, IL-1β, and GSDMD, reduced levels of IL-1β and GSDMD, decreased cell apoptosis, and weakened ROS and NLRP3 fluorescence intensity; compared to the Siglec5-OE + Rocaglamide+Piceatannol+mSIRK+TDE group, the Siglec5-OE + Rocaglamide+Piceatannol+mSIRK+TDE + SIRP*α*-OE group showed increased protein expression of NLRP3, IL-1β, and GSDMD (Figure 9A), elevated levels of IL-1β and GSDMD (Figure 9B), significantly increased cell apoptosis (Figure 9C), and enhanced ROS and NLRP3 fluorescence intensity (Figures 9D,E).

## 4 Discussion

Sepsis and its associated lung injury represent a complex pathological process involving interactions among multiple molecules and signaling pathways. This study, through *in vivo* and *in vitro* experiments, provides evidence suggesting a protective role of Siglec-5 in LPS-induced lung injury and proposes a working model for its potential molecular mechanisms.

In sepsis, the release of damage-associated molecular patterns (DAMPs) is a critical step in triggering inflammatory responses (13). DAMPs activate downstream signaling pathways by binding to pattern recognition receptors (PRRs), with spleen tyrosine kinase (SYK) playing a significant role in this process (14). Studies have demonstrated that damage-associated molecular patterns (DAMPs), including high-mobility group box 1 (HMGB1) and heat shock proteins (HSPs), induce SYK phosphorylation by engaging receptors such as Dectin-1 and Dectin-3 on macrophage surfaces (15). Conventionally, Signal Regulatory Protein α (SIRPα) is characterized as an inhibitory receptor that acts as a checkpoint to dampen immune activation and maintain homeostasis (16, 17). However, our findings reveal a paradoxical role in LPS-induced lung injury, where SIRPα overexpression exacerbates tissue damage rather than alleviating it. We propose that this shift from a protective “brake” to a pathological driver is mediated by the SYK-SHP2-ERK1/2-ELK1 positive feedback loop identified in this study. In the hyperinflammatory environment of sepsis, this loop drives the excessive transcriptional upregulation of SIRPα. This signal saturation effectively overrides the receptor’s physiological inhibitory capacity, leading to sustained ERK1/2 activation and amplified ROS production. Consequently, SIRPα contributes to the maladaptive immune response and tissue injury (18).

ROS production serves as a central mechanism underlying LPS-induced lung injury. Beyond causing direct oxidative damage to cell membranes and DNA, ROS activates the NLRP3 inflammasome. Activation of the NLRP3 inflammasome triggers the proteolytic maturation and secretion, such as IL-1β and IL-18, thereby further amplifying the inflammatory cascade (19). Pyroptosis, a form of programmed cell death characterized by cell membrane rupture and inflammatory factor release, further aggravates tissue damage. This study found that Siglec-5 enhances the synthesis of heat shock factor 1 (HSF1), increasing the expression of heat shock protein 90 (Hsp90) and heat shock protein 70 (Hsp70) (20). Hsp90 and Hsp70 have antioxidant stress properties that prevent the generation of ROS, which lowers pyroptosis and NLRP3 inflammasome activation (21). A notable finding is the potential protein association between the membrane receptor Siglec-5 and the intracellular/nuclear factor HSF1. While Siglec-5 is traditionally a surface receptor, our Co-IP data indicate a possible functional association rather than a confirmed direct interaction. While Siglec-5 is traditionally a surface receptor, our Co-IP data suggests a functional association. We propose that Siglec-5 signaling may stabilize HSF1 protein expression—potentially by inhibiting its ubiquitin-proteasome degradation or by facilitating its nuclear translocation—thus preventing the oxidative collapse of the cell. Furthermore, Siglec-5 exerts its protective effect by suppressing the phosphorylation of SYK and ERK1/2. This inhibition is crucial as it disrupts the maladaptive positive feedback loop that drives the transcriptional overexpression of SIRPα. By blocking this axis, Siglec-5 prevents the “SIRPα paradox”—where excessive SIRPα exacerbates injury—and effectively reduces the inflammatory cascade and tissue damage. According to this research, Siglec-5 prevents SIRPα transcriptional expression by inhibiting ERK1/2 phosphorylation. This finding sheds fresh light on Siglec-5’s function in sepsis. Future research could further explore the interaction between Siglec-5 and SIRPα and its specific mechanisms in LPS-induced lung injury.

**Figure 10:**
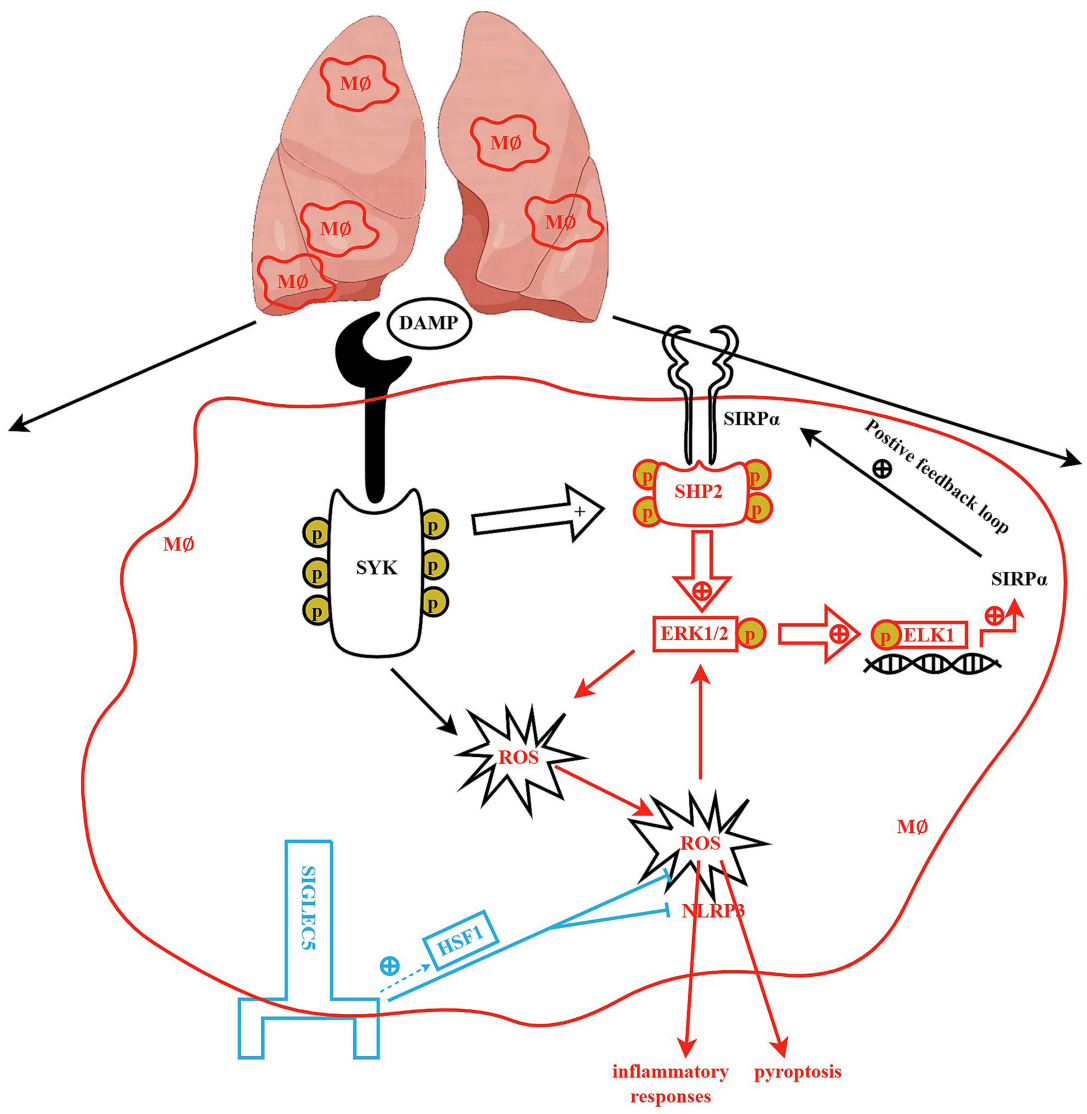
Siglec-5 inhibits LPS-induced lung injury by modulating the HSF1/SYK/ERK1/2/SIRPα signaling pathway to suppress ROS and pyroptosis responses.

The findings of this study provide new theoretical foundations and potential therapeutic targets for LPS-induced lung injury. Based on our data, we propose a working model in which Siglec-5, by modulating the HSF1/SYK/ERK1/2/SIRPα axis, inhibits ROS production and pyroptosis, thereby alleviating inflammatory responses and tissue damage (Figure 10). Future research could further validate Siglec-5 expression in clinical samples and its relationship with sepsis patient prognosis, offering more evidence for its clinical application. Additionally, developing drugs targeting Siglec-5 and its related signaling pathways may provide new strategies for treating LPS-induced lung injury. However, we acknowledge that the proposed pathway is complex and several steps are inferred from literature or inhibitor studies rather than direct molecular evidence; no temporal or epistasis analyses were performed to establish hierarchical order. Therefore, this signaling cascade should be considered a proposed working model requiring further experimental validation. We acknowledge potential species differences between mouse and human Siglec-5, but this study serves as a proof-of-concept in a relevant murine model. Furthermore, the intraperitoneal LPS injection model represents endotoxemia rather than true polymicrobial sepsis, and no survival analysis or physiological/organ function assessments were performed; therefore, caution is needed when extrapolating these results to clinical sepsis.

## Data Availability

The raw data supporting the conclusions of this article will be made available by the authors, without undue reservation.
