# Supplementary figures and images for: Siglec-5 suppresses LPS-induced acute lung injury via negative regulation of HSF1/SYK-mediated ROS production and pyroptosis
Source: Front Med (Lausanne). 2026 Jun 30;13:1799874. doi: 10.3389/fmed.2026.1799874 (PMC13365134; doi:10.3389/fmed.2026.1799874)

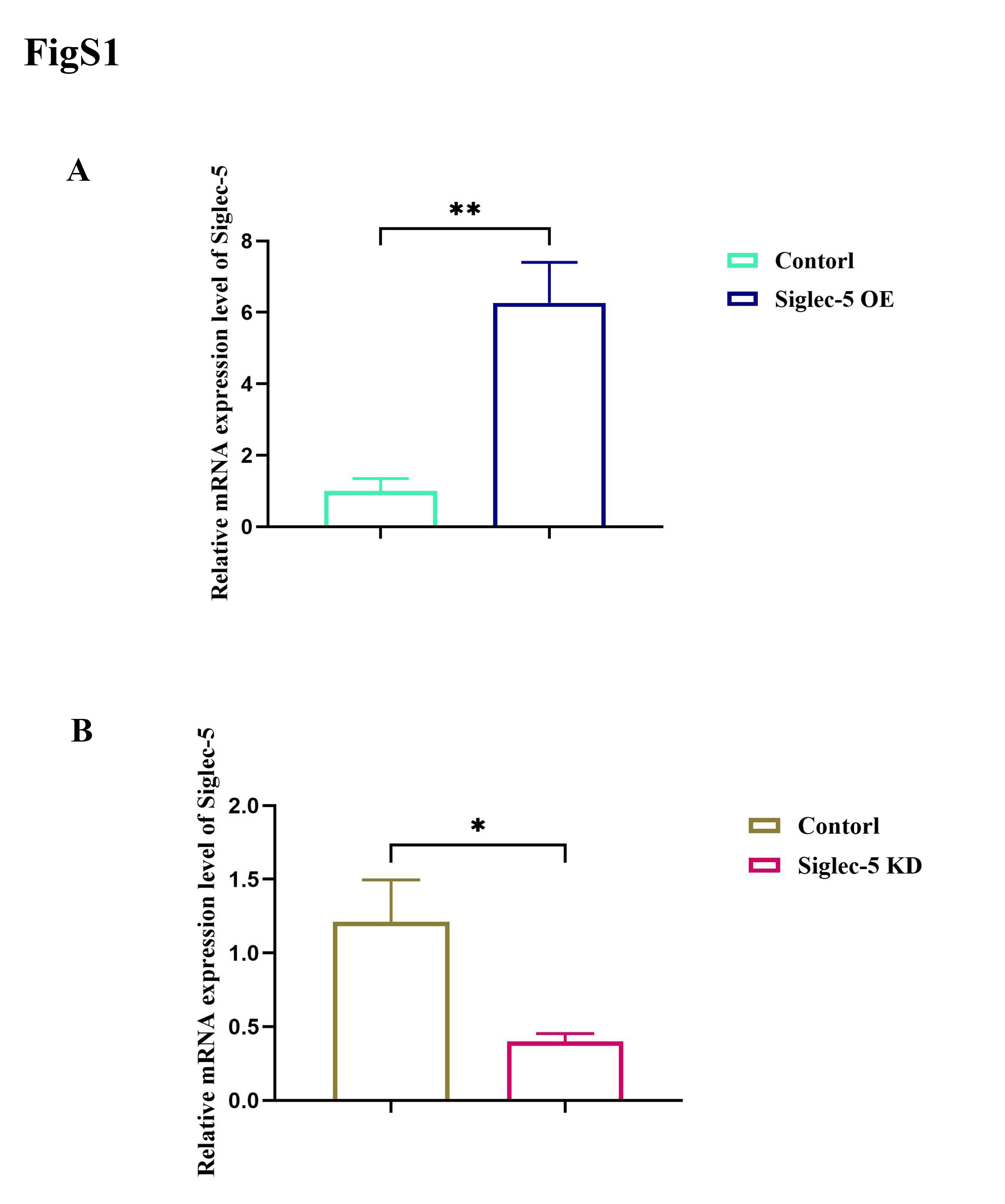

Supplement: Supplementary file 1 [file Image_1.jpg]
