# Supplementary material for: Siglec-5 suppresses LPS-induced acute lung injury via negative regulation of HSF1/SYK-mediated ROS production and pyroptosis
Source: Front Med (Lausanne). 2026 Jun 30;13:1799874. doi: 10.3389/fmed.2026.1799874 (PMC13365134; doi:10.3389/fmed.2026.1799874)
Supplement: Supplementary file 2 [file Supplementary_File_1.docx]

Supplementary File 1.

Primers applied to quantitative real-time PCR.

| Primers | Sequence(5'->3') |  |
| --- | --- | --- |
| NLRP3 | Forward | 5'-ATTACCCGCCCGAGAAAGG-3' |
|  | Reverse | 5'-TCGCAGCAAAGATCCACACAG-3' |
| SYK | Forward | 5′-CCAACCACCTGACCTACTTTTT-3′ |
|  | Reverse | 5′-ATTAAGTTCCCTCGATGGTG-3′ |
| GSDMD | Forward | 5′-GAGTGTCCTAGAGCTGG-3′ |
|  | Reverse | 5′-GGCTCAGTCCTGATAGCAGTG-3′ |
| Siglec-5 | Forward | 5'-TTCAGGAACGGCATAGCCCTA-3' |
|  | Reverse | 5'-TACTCGACGAAGCTCCAAGAT-3' |
| GAPDH | Forward | 5'-GCCTCAAAATCCTCTCGTTGTG-3' |
|  | Reverse | 5'-GGAAGATGGTGATGGGATTTC-3' |
